# A245 CROHN’S DISEASE-ASSOCIATED BACTERIAL PATHOBIONT EVOKED EPITHELIAL MITOCHONDRIAL DAMAGE DRIVES TYPE I INTERFERON EXPRESSION

**DOI:** 10.1093/jcag/gwad061.245

**Published:** 2024-02-14

**Authors:** A Mohan, A Wang, D McKay, T Shutt

**Affiliations:** University of Calgary, Calgary, AB, Canada; University of Calgary, Calgary, AB, Canada; University of Calgary, Calgary, AB, Canada; University of Calgary, Calgary, AB, Canada

## Abstract

**Background:**

The Crohn’s disease-associated adherent invasive *E. coli* (AIEC) (strain LF82) decreases mitochondrial membrane potential and fragments the epithelial mitochondrial network. Due to the evolutionary history of mitochondria as former bacteria, they present a variety of molecular patterns known to trigger innate immune responses. Specifically, mitochondrial DNA (mtDNA) released following mitochondrial damage causes upregulation of type I interferons, priming inflammatory signalling. Thus, we hypothesized that AIEC-induced mitochondrial dysfunction results in mtDNA release to activate innate immune signalling that could contribute to disease pathogenesis in the gut.

**Aims:**

We aimed determine if an inflammatory response occurs downstream of mitochondrial damage evoked by *E. coli*-LF82 infection.

**Methods:**

Control T84 epithelial cells, T84s infected *E. coli*-LF82 (10^8^ cfu, MOI=100, 4h) and T84s infected with the commensal *E. coli*-HB101 (10^8^ cfu for an MOI=100, 4h) were collected for protein and RNA extraction. RNA was used to measure IFNα and IFNβ expression following infection, while protein was used to measure upstream mediators including phosphorylation of TANK Binding Kinase-1 (TBK1) via Western blot.

**Results:**

Epithelia infected with *E. coli*-LF82 but not *E. coli*-HB101 displayed dramatic fragmentation of their mitochondria network and increased levels of phosphorylated-TBK1 compared to control T84 cells (pampersand:003C0.01). Assessment of IFNα and IFNβ mRNA revealed significant up-regulation *E. coli*-LF82 infected T84 cells and human colon organoids.

**Conclusions:**

Our findings identify a novel role for *E. coli*-LF82 in activating type I interferon response and for the first time show AIEC-induced activation of TBK1 and type I interferons, which may occur through mitochondrial damage, rather than through response to bacterial molecular patterns. As a result, our findings suggest that the AIEC colonization seen in Crohn’s disease may dysregulate inflammatory signalling through mitochondrial damage. Ultimately, we believe this dysregulation of inflammatory signalling may sensitize the host to further inflammatory signalling, contributing to the pathogenesis of inflammatory bowel disease.

**Funding Agencies:**

CIHR

